# Supplementary material for: Bacteria are a major determinant of Orsay virus transmission and infection in Caenorhabditis elegans
Source: eLife. 2024 Jul 11;12:RP92534. doi: 10.7554/eLife.92534 (PMC11239179; doi:10.7554/eLife.92534)
Supplement: Supplementary file 1. [file elife-92534-supp1.docx]

## Supplementary Information

#### Supplementary Table 1: List of *Caenorhabditis elegans* strains used in this study.

| **Strain Name** | **Genotype** | **Source** |
| --- | --- | --- |
| WUM29 | *virIs1[pHIP::Orsay-RNA1;pHIP::Orsay-RNA2;myo-2p::YFP];rde-1(ne219);jyIs8(pasl-5p::gfp;myo-2p::mCherry)* | Gift of Wang lab^1^ |
| ERT54 | *jyIs8[pals-5p::GFP;myo-2p::mCherry]* | CGC |
| WUM31 | *rde-1(ne219);jyIs8[pals-5p::GFP;myo-2p::mCherry]* | Gift of Wang lab^2^ |
| SS104 | *glp-4(bn-2)* | CGC |
| ZD2610 | *rde-1(ne219);jyIs8[pals-5p::GFP;myo-2p::mCherry]; glp-4(bn-2)* | This study |
| ZD2611 | *jyIs8[pals-5p::GFP;myo-2p::mCherry]; glp-4(bn-2)* | This study |
| WUM104 | *virEx[pHIP::RNA1WT-1]* | Gift of Wang lab^2^ |
| WUM106 | *virEx[pHIP::RNA1D601A-1]* | Gift of Wang lab^2^ |

#### Supplementary Table 2: List of bacterial strains used in this study.

| **Strain Name** | **Genotype** | **Source** |
| --- | --- | --- |
| *Escherichia coli OP50* | WT | Kim Lab |
| *Enterobacter xiangfangensis CEN2ent1* | WT | CGC^3^ |
| *Lelliottia amnigena JUb66* | WT | CGC^3^ |
| *Pseudomonas mendocina MSPm1* | WT | CGC^3^ |
| *Comamonas piscis BIGb0172* | WT | CGC^3^ |
| *Pantoea sp. BIGb0393* | WT | CGC^3^ |
| *Providencia sp. JUb39* | WT | Gift of Félix Lab^4^ |
| *Klebsiella sp.* J*Ub268* | WT | Gift of Félix Lab – unpublished |
| *Achromobacter sp. JUb24* | WT | Gift of Félix Lab^4^ |
| *Ochrobactrum vermis MYb71* | WT | CGC^3^ |
| *Ochrobactrum BH3* | WT | Gift of Troemel Lab^5^ |
| *GFP + Ochrobactrum BH3* | WT(GFP+) | Gift of Troemel Lab^5^ |
| *Pseudomonas lurida MYb11* | WT | CGC^3^ |
| *Pseudomonas lurida MYb11* | ΔgacA | This study |
| *Pseudomonas lurida MYb11* | ΔkinB | This study |
| *Pseudomonas lurida MYb11* | ΔprpC | This study |
| *Pseudomonas lurida MYb11* | ΔptsP | This study |
| *Pseudomonas aeruginosa PA01* | WT | Gift of Dove Lab |
| *Pseudomonas aeruginosa PA01* | ΔlasI | Gift of Greenberg Lab^6^ |
| *Pseudomonas aeruginosa PA01* | ΔlasR | Gift of Greenberg Lab^6^ |
| *Pseudomonas aeruginosa PA01* | ΔrhlI | Gift of Greenberg Lab^6^ |
| *Pseudomonas aeruginosa PA01* | ΔrhlR | Gift of Greenberg Lab^6^ |
| *Pseudomonas aeruginosa PA01* | ΔpqsA | Gift of Dove Lab - unpublished |
| *Pseudomonas aeruginosa PA01* | ΔgacA | Gift of Dove Lab^7^ |
| *Pseudomonas aeruginosa PA01* | Δpel | Gift of Parsek Lab^8^ |
| *Pseudomonas aeruginosa PA01* | Δpsl | Gift of Parsek Lab^9^ |
| *Pseudomonas aeruginosa PA01* | ΔalgD | Gift of Parsek Lab^10^ |
| *Pseudomonas aeruginosa PA14* | WT | Kim Lab |
| *Pseudomonas aeruginosa PA14* | ΔlasI | Gift of E. Drenkard and Ausubel Lab – Not published |
| *Pseudomonas aeruginosa PA14* | ΔlasR | Gift of E. Drenkard and Ausubel Lab^11^ |
| *Pseudomonas aeruginosa PA14* | ΔrhlI | Gift of E. Drenkard and Ausubel Lab^12^ |
| *Pseudomonas aeruginosa PA14* | ΔrhlR | Gift of E. Drenkard and Ausubel Lab^11^ |
| *Pseudomonas aeruginosa PA14* | ΔpqsE | Gift of Paczkowski Lab^13^ |
| *Pseudomonas aeruginosa PA14* | ΔrhlI;ΔpqsE | Gift of Paczkowski Lab^13^ |
| *Pseudomonas aeruginosa PA14* | ΔgacA | Gift of Pukkila-Worley Lab^14^ |
| *Pseudomonas aeruginosa PA14* | ΔpelA | Gift of E. Drenkard and Ausubel Lab^15^ |
| *Pseudomonas aeruginosa PA14* | ΔalgD | Gift of E. Drenkard and Ausubel Lab^16^ |
| *Pseudomonas aeruginosa PA14* | Mutants from the Nonredundant PA14 Transposon Insertion Library | E. Drenkard and Ausubel Lab^14^ |

#### Supplementary Table 3: List of *P. aeruginosa* PA14 transposon insertion mutants screened in this study.

## Supplementary References

1. Jiang, H., Franz, C. J. & Wang, D. Engineering Recombinant Orsay Virus Directly in the Metazoan Host Caenorhabditis elegans. *J Virol* **88**, 11774–11781 (2014).

2. Jiang, H., Chen, K., Sandoval, L. E., Leung, C. & Wang, D. An Evolutionarily Conserved Pathway Essential for Orsay Virus Infection of *Caenorhabditis elegans*. *mBio* **8**, (2017).

3. Dirksen, P. *et al.* CeMbio - The *Caenorhabditis elegans* Microbiome Resource. *G3 Genes|Genomes|Genetics* **10**, 3025–3039 (2020).

4. Samuel, B. S., Rowedder, H., Braendle, C., Félix, M.-A. & Ruvkun, G. *Caenorhabditis elegans* responses to bacteria from its natural habitats. *Proceedings of the National Academy of Sciences* **113**, (2016).

5. Troemel, E. R., Félix, M.-A., Whiteman, N. K., Barrière, A. & Ausubel, F. M. Microsporidia Are Natural Intracellular Parasites of the Nematode Caenorhabditis elegans. *PLoS Biol* **6**, e309 (2008).

6. Wang, M., Schaefer, A. L., Dandekar, A. A. & Greenberg, E. P. Quorum sensing and policing of *Pseudomonas aeruginosa* social cheaters. *Proceedings of the National Academy of Sciences* **112**, 2187–2191 (2015).

7. Brencic, A. *et al.* The GacS/GacA signal transduction system of *Pseudomonas aeruginosa* acts exclusively through its control over the transcription of the RsmY and RsmZ regulatory small RNAs. *Mol Microbiol* **73**, 434–445 (2009).

8. Starkey, M. *et al.* *Pseudomonas aeruginosa* Rugose Small-Colony Variants Have Adaptations That Likely Promote Persistence in the Cystic Fibrosis Lung. *J Bacteriol* **191**, 3492–3503 (2009).

9. Kirisits, M. J., Prost, L., Starkey, M. & Parsek, M. R. Characterization of Colony Morphology Variants Isolated from *Pseudomonas aeruginosa* Biofilms. *Appl Environ Microbiol* **71**, 4809–4821 (2005).

10. Wozniak, D. J. *et al.* Alginate is not a significant component of the extracellular polysaccharide matrix of PA14 and PAO1 *Pseudomonas aeruginosa* biofilms. *Proceedings of the National Academy of Sciences* **100**, 7907–7912 (2003).

11. Limmer, S. *et al.* *Pseudomonas aeruginosa* RhlR is required to neutralize the cellular immune response in a *Drosophila melanogaster* oral infection model. *Proceedings of the National Academy of Sciences* **108**, 17378–17383 (2011).

12. Haller, S. *et al.* Quorum‐sensing regulator RhlR but not its autoinducer RhlI enables *Pseudomonas* to evade opsonization. *EMBO Rep* **19**, (2018).

13. Simanek, K. A. *et al.* The PqsE-RhlR Interaction Regulates RhlR DNA Binding to Control Virulence Factor Production in *Pseudomonas aeruginosa*. *Microbiol Spectr* **10**, (2022).

14. Liberati, N. T. *et al.* An ordered, nonredundant library of *Pseudomonas aeruginosa* strain PA14 transposon insertion mutants. *Proceedings of the National Academy of Sciences* **103**, 2833–2838 (2006).

15. Friedman, L. & Kolter, R. Genes involved in matrix formation in Pseudomonas aeruginosa PA14 biofilms. *Mol Microbiol* **51**, 675–690 (2003).

16. Yorgey, P., Rahme, L. G., Tan, M. W. & Ausubel, F. M. The roles of mucD and alginate in the virulence of Pseudomonas aeruginosa in plants, nematodes and mice. *Mol Microbiol* **41**, 1063–76 (2001).
